# FocusStack and StimServer: a new open source MATLAB toolchain for visual stimulation and analysis of two-photon calcium neuronal imaging data

**DOI:** 10.3389/fninf.2014.00085

**Published:** 2015-01-20

**Authors:** Dylan R. Muir, Björn M. Kampa

**Affiliations:** ^1^Department of Neurophysiology, Brain Research Institute, University of ZürichZürich, Switzerland; ^2^Biozentrum, University of BaselBasel, Switzerland; ^3^Department of Neurophysiology, Institute of Biology 2, RWTH Aachen UniversityAachen, Germany

**Keywords:** two-photon calcium imaging, neuronal responses, Matlab, visual stimulus generation, analysis toolbox, small memory footprint, open source

## Abstract

Two-photon calcium imaging of neuronal responses is an increasingly accessible technology for probing population responses in cortex at single cell resolution, and with reasonable and improving temporal resolution. However, analysis of two-photon data is usually performed using *ad-hoc* solutions. To date, no publicly available software exists for straightforward analysis of stimulus-triggered two-photon imaging experiments. In addition, the increasing data rates of two-photon acquisition systems imply increasing cost of computing hardware required for in-memory analysis. Here we present a Matlab toolbox, FocusStack, for simple and efficient analysis of two-photon calcium imaging stacks on consumer-level hardware, with minimal memory footprint. We also present a Matlab toolbox, StimServer, for generation and sequencing of visual stimuli, designed to be triggered over a network link from a two-photon acquisition system. FocusStack is compatible out of the box with several existing two-photon acquisition systems, and is simple to adapt to arbitrary binary file formats. Analysis tools such as stack alignment for movement correction, automated cell detection and peri-stimulus time histograms are already provided, and further tools can be easily incorporated. Both packages are available as publicly-accessible source-code repositories^1^.

## 1. Introduction

Two-photon calcium imaging has become a major method to record neuronal activity. However, analysis of the acquired data has special requirements because of the image based data format. In addition, increasing spatial and temporal resolution also require increasing computation power of the analysis system. While consumer computing hardware is cheap and accessible for most researchers, it is usually limited in maximum addressable memory. Microscopes with resonance scanners, which are increasingly becoming standard equipment for two-photon imaging of neuronal signals on fast timescales, can easily generate in the order of 10 MB (10 × 2^20^ bytes) of data per second. Coupled with the trend toward imaging in awake, behaving animals, which necessitates lengthly imaging trials, single imaging sessions can produce 10 of gigabytes (10 × 2^30^ bytes) of data. In-memory analysis of two-photon imaging data entails considerable hardware requirements (and increasingly so), leading to a rapid rise in cost and accessibility.

Here we present a new, open-source, Matlab-based two-photon analysis toolchain designed to process large two-photon imaging stacks with only a small memory footprint. This makes analysis possible on standard consumer hardware. We also present a new open-source Matlab-based server for visual stimulus generation and presentation which can be controlled remotely over TCP or UDP network links.

In Section 2 we present a high-level overview of our stimulation and analysis toolchain. In Section 3 we discuss how the end user interacts with FocusStack, and how the design of FocusStack makes analysis of two-photon imaging data simpler. In Section 4 we present the low-level representation of a FocusStack object, and discuss how FocusStack can easily be adapted to new two-photon imaging data formats. In Section 5 we discuss the design of StimServer, and how stimuli are configured and queued during an experiment. In Section 6 we present an example two-photon imaging experiment and analysis using StimServer and FocusStack. The experimental data analyzed, and Matlab scripts required to reproduce the analysis, are available as Supplementary Material.

### 1.1. Existing two-photon stack analysis packages

The only other publicly-available two-photon processing system, at time of writing, is the Two-Photon Processor (2PP; Tomek et al., 2013). 2PP is a GUI-based Matlab toolbox for analyzing two-photon calcium data, and performs automated ROI segmentation, stack alignment, and calcium signal extraction. Our toolchain differs from 2PP in a number of ways:

- FocusStack is aware of stimulus identity and timing, and automates derandomization of time-series data, when stimuli are presented in random order;
- FocusStack is command-based, as opposed to the GUI-based interface of 2PP. We believe a command-based interface is more appropriate for analysis of experimental data, where identical analysis steps need to be repeated for several data sets;
- FocusStack is designed for small memory requirements, using novel Matlab classes for efficient data access. 2PP is not designed for lazy data access, implying that entire stacks must be analyzed in-memory, with a consequently large memory footprint;
- FocusStack interfaces directly with additional Matlab analysis tools for spike estimation from calcium response traces. 2PP incorporates these algorithms internally.

## 2. Toolchain overview

The design goal of FocusStack and StimServer was to provide simple, extensible tools to assist experiments using two-photon calcium imaging of neuronal responses; accessible to those with little programming experience, but powerful enough to automate most low-level analysis of calcium response stacks. Although alternative programming languages are increasing in popularity (such as Python, R and Octave), Matlab remains an accessible and frequently used tool for analysis and statistical testing, with a reputation for simple uptake by non-programmers. For this reason, we designed a toolchain that does allows users to design and script their entire analysis in Matlab, without the need for additional software packages.

**Figure 1:**
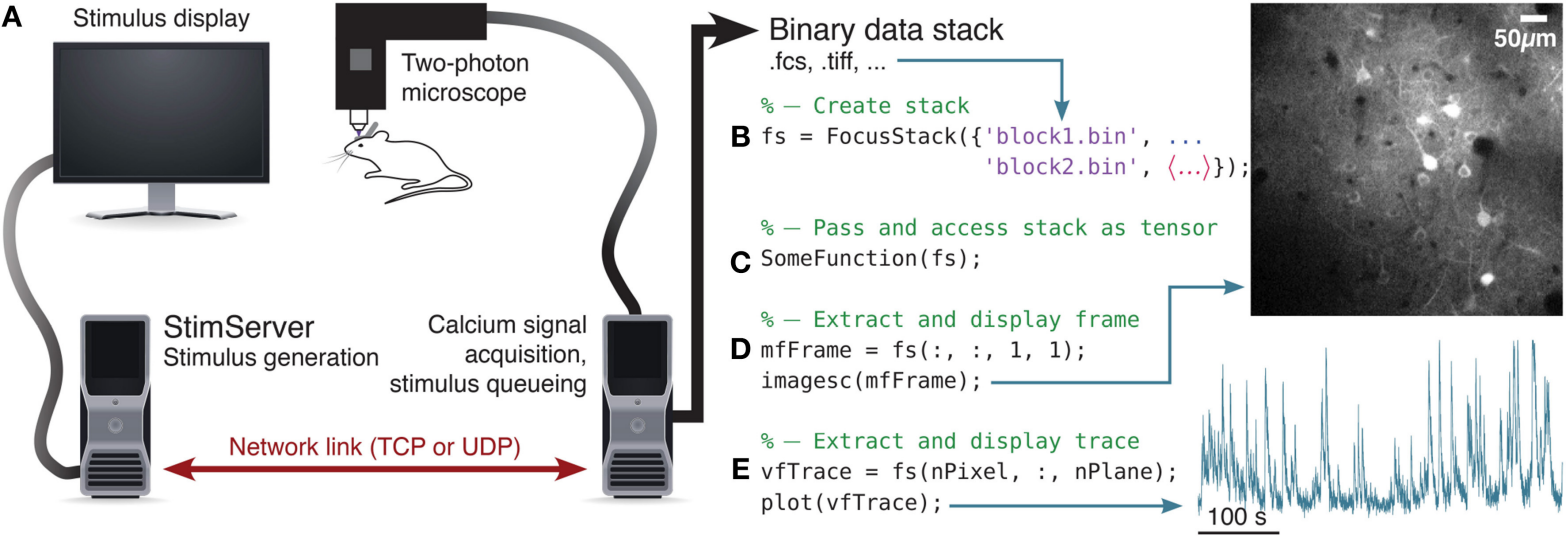
**Overview of visual stimulation, stack acquisition and analysis in Matlab. (A)** StimServer is used to generate and present visual stimuli to an animal, under the control of a calcium imaging system, over a network link. Data is stored in a binary format. **(B)** A FocusStack object is created in Matlab, to access several sequential data blocks as a single concatenated stack. **(C)** The FocusStack object “fs” can now be accessed as a Matlab tensor, and passed into Matlab functions. **(D)** Extracting a single frame is as simple as referencing a Matlab tensor. **(E)** Extracting the response trace through time of a single pixel is equally simple.

An overview of a two-photon acquisition and visual stimulation setup is shown in Figure 1. Due to the real-time requirements of both visual stimulation and acquisition of two-photon imaging data, these tasks are usually performed on separate dedicated computing systems (Figure 1A). StimServer is controlled over a TCP or UDP network link, to trigger stimulus presentation and sequencing. Two-photon acquisition occurs using the software appropriate for the experimental equipment used, and stores the resulting imaging stacks as binary data files on disk. Ideally, meta-data about the stack—stimulus identity and random sequencing, stack resolution, information about the acquisition system, etc.—are stored with the stack data files in a file header or a “side-car” meta-data file.

Binary stack data files are analyzed in Matlab, by using FocusStack to map several stack files to a single FocusStack object (Figure 1B). This object appears as a simple Matlab tensor (i.e., a multi-dimensional Matlab matrix), with frames, channels, and single pixels accessed using standard Matlab referencing (Figures 1D-E). Since FocusStack objects can be accessed as Matlab tensors, many existing Matlab analysis functions that expect tensors can seamlessly be passed FocusStack objects without modification (Figure 1C).

However, FocusStack objects are aware of stimulus timing and sequencing, provide services for stack alignment, provide support for assigning baseline fluorescence distributions, and have simple helper functions to perform derandomization and segmentation of stack data. These facilities are described in the next section. An example flow-chart for analysis using a FocusStack object is shown in Figure 3.

## 3. High-level interface to FocusStack

The design of FocusStack is to provide a smart wrapper interface to two-photon imaging data. An example of creating and accessing a two-photon imaging stack using a FocusStack object is given in Figure 1B. Several files of acquired calcium responses can be wrapped by a FocusStack object, to appear to Matlab and to calling functions as a simple Matlab tensor (Figures 1C–E).

### 3.1. Stack meta-data

**Figure 2:**
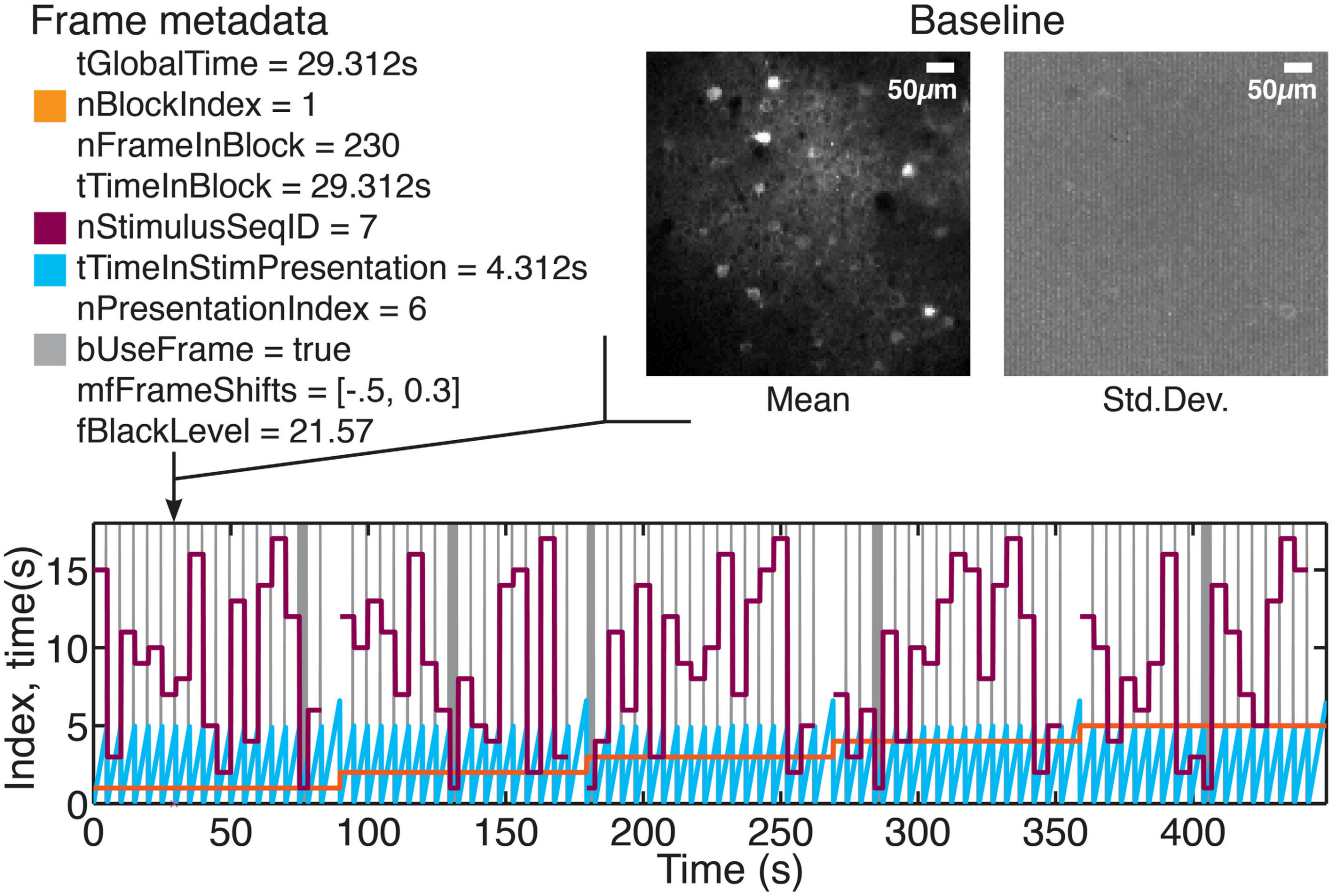
**Stimulus information and other meta-data associated with each frame**. A series of drifting grating stimuli were presented in randomized order (see values of nStimulusSeqID), over several repeats (see values of nBlockIndex). Using FocusStack.FrameStimulusInfo, the stimulus meta-data associated with each frame can be accessed (meta-data is listed for the frame indicated by the arrow). In addition, each frame is associated with a mis-alignment shift (mfFrameShifts), a “black” level (vfBlackTrace), and a per-pixel baseline distribution (insets at top right). Colors at top left indicate the corresponding traces of meta-data values in the time-series plot.

Although a FocusStack object can be accessed just like a Matlab tensor, each frame and pixel in the stack has many items of meta-data associated with it (see Figure 2 and Tables 1,2). Meta-data consists of stack-global information such as the resolution (pixels per μm) and dimensions of the stack, imaging frame rate and Z-step per frame. Stimulus-specific global meta data includes the number of unique stimuli presented, the duration of each stimulus, the order of presentation, and which periods of the stimulus presentation should be used for analysis (see Listing 2).

**Table 1:** **List of meta-data provided by a FocusStack object**.

**Meta-data parameter name**	**Contents**
**STACK-GLOBAL META-DATA**	
.fPixelsPerUM	Spatial calibration of the stack (X and Y), in pixels per μm
.tFrameDuration	Acquisition time per frame, in seconds
.fZStep	Z spacing between subsequent frames, in μm
.mfFrameShifts	Misalignment shifts for each frame, in fractional pixels. Assigned manually, or using alignment method FocusStack/Align
.vfBlackTrace	Black set-point value for each frame, in raw units. Assigned manually, or using utility method FocusStack/DefineBlackRegion
**STIMULUS-RELATED META-DATA**	
.tBlankTime	Blank time between episodic visual stimuli
.vnStimulusIDs	List of stimuli presented (one stimulus ID per data file). Each stimulus ID can contain a sequence of several individual stimuli
.nNumStimuli	(Read-only)Total number of individual stimulus sequence IDs presented in the entire stack
.cvnSequenceIDs	Cell array, each element containing a vector of stimulus sequence IDs in the order they were presented
.vtStimulusDurations	Vector of stimulus sequence ID durations, in seconds. Each entry specifies the duration of the corresponding stimulus sequence ID
.vtStimulusStartTimes	Vector of onset times for each individual stimulus presentation, as an offset in seconds from the first frame of the stack. Assigned manually, or computed automatically
.vtStimulusEndTimes	Vector of end times for each individual stimulus presentation, as an offset in seconds from the first frame of the stack. Assigned manually, or computed automatically
.mtStimulusUseTimes	Matrix of times indicating which periods of stimulus presentation should be used for analysis. Each row corresponds to a stimulus sequence ID, and is a row vector [tStartTime tStopTime], indicating offsets from the start of the presentation of the corresponding stimulus

All the parameters listed here are FocusStack class properties, and should be assigned from meta-data stored with the data file, whenever possible.

**Table 2:** **List of meta-data provided by a FocusStack object (continued from Table 1)**.

**Meta-data parameter name**	**Contents**
**FRAME-RELATED META-DATA**	
vtGlobalTime	The time in seconds since the first frame in the stack. (FSI)
vnBlockIndex	The index of the block (data file) the associated frame falls within. (FSI)
vnFrameInBlock	The index of the associated frame within the block, with the first frame given index 1. (FSI)
vtTimeInBlock	The time in seconds since the first frame in the block. (FSI)
vnStimulusSeqID	The stimulus sequence ID associated with each frame. (FSI)
vtTimeInStim Presentation	The time in seconds of the associated frame since the onset of the stimulus in which the frame falls. (FSI)
vnPresentationIndex	The index of the current stimulus presentation in the entire stack. The first stimulus is given index 1. (FSI)
vbUseFrame	A boolean value associated with each frame, indicating whether that frame should be used for analysis. (FSI)
tfBlankMean, tfBlankStd	Mean and standard deviation distribution of the baseline distribution assigned to a stack. Obtained by referencing a stack using fs.BlankFrames(<stack reference>) or by using the FocusStack/GetCorresponding BlankFrames class method
	The baseline distribution is assigned using the FocusStack/AssignBlankFrame class method (see Section 3.1.2 and Listing 1)

Parameters listed that specify “FSI” for access are obtained using the FocusStack/FrameStimulusInfo class method, and are computed from the parameters given in Table 1.

Associating this meta-data with the stack enables FocusStack to provide detailed information about each frame (Figure 2), using the FrameStimulusInfo method. Each frame is tagged with the information about the stimulus being presented while that frame was being acquired.

For file formats supported out of the box by FocusStack, meta-data stored in the data files is automatically loaded and assigned to the FocusStack object. When implementing two-photon acquisition software and extending FocusStack to support the corresponding file formats, care should be taken to store and automatically load as much meta-data as possible.

#### 3.1.1. Stack alignment

Ensuring that each successive frame is aligned with the previous frames in the stack is essential for extracting calcium responses with high signal to noise ratio. FocusStack provides transparent internal support for sub-pixel rigid linear translation of each frame. Each frame in a FocusStack object has a [2 × *N*] double list mfFrameShifts of displacements for each frame, relative to the unshifted stack origin. If misalignments are assigned to the stack, then FocusStack transparently shifts each frame when the stack is accessed. Re-alignment of each frame occurs lazily, the first time a frame is requested, and is subsequently cached on disk for quick repeated access.

FocusStack includes a sub-pixel, rigid linear translation alignment algorithm based on Fourier phase matching (Guizar-Sicairos et al., 2008; example in Listing 1). This algorithm supports progressive alignment or alignment to a reference frame or image. Per-frame spatial filtering, sliding-window frame averaging and single-frame shift size rejection to regularize the alignment process. However, any alignment algorithm can be used and the shifts manually assigned to the FocusStack.mfFrameShifts property.

**Listing 1:**
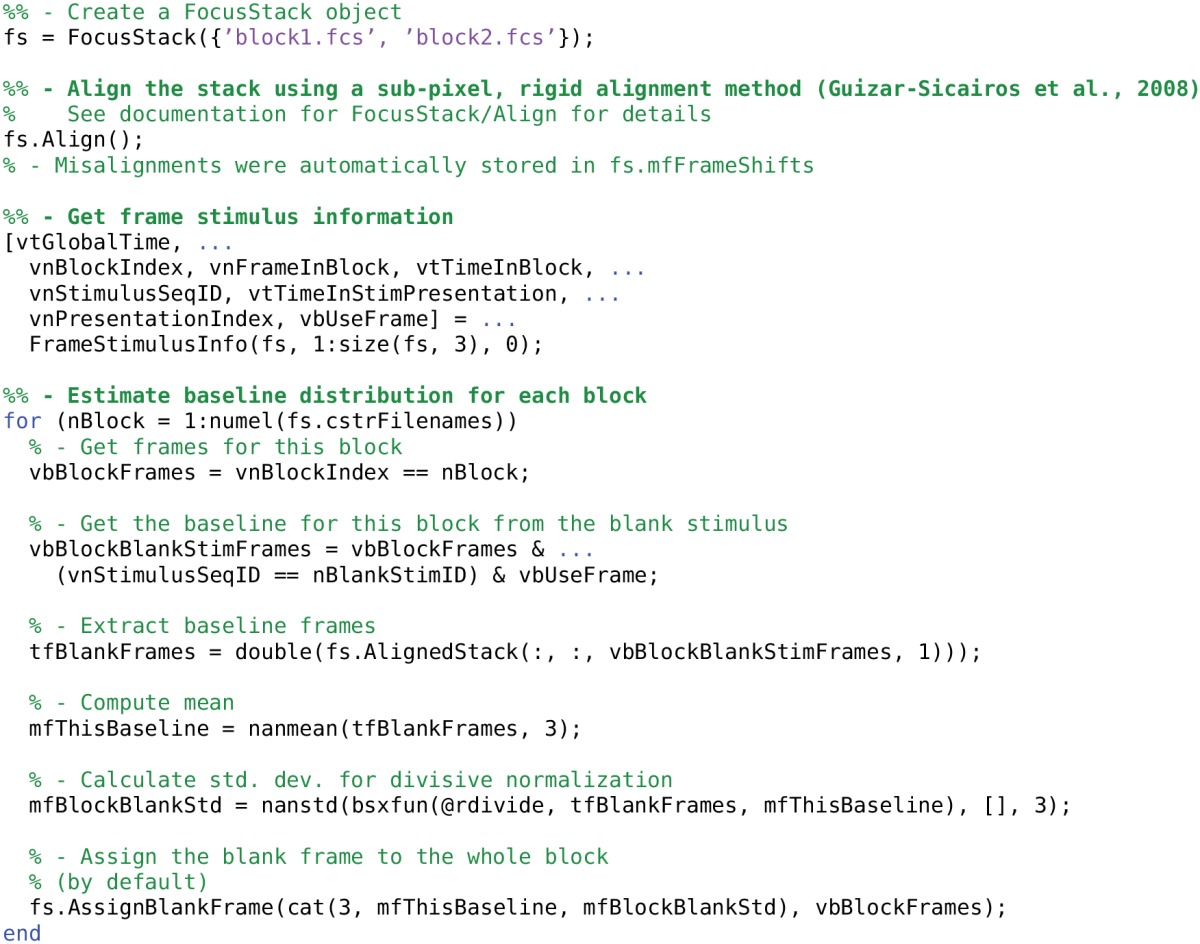
**Creating a FocusStack object; performing stack alignment; estimating the baseline fluorescence distribution and assigning it to the FocusStack object**.

#### 3.1.2. Defining the baseline fluorescence distribution

Two-photon calcium imaging commonly uses fluorescent dyes that change their conformation and emission properties when bound to Ca^2+^ molecules. In single-channel imaging, the calcium concentration change (a proxy for firing rate) of a neuron is related to the proportional increase in fluorescence (e.g., OGB; Grynkiewicz et al., 1985). In FRET imaging, a molecule consisting of two bound fluorophores with differing emission wavelengths causes a differential change in fluorescence of both fluorophores when bound to Ca^2+^ (e.g., Yellow Cameleon; Nagai et al., 2004). In this case the response signal is the ratio of the responses in two imaging channels.

Both of these techniques require estimation of the “baseline” fluorescence (*F*_0_) of a neuron to define the differential calcium response Δ*F*/*F*_0_. In FocusStack, this is obtained through two mechanisms. Firstly, each frame is assigned a “black” reference level using the FocusStack.DefineBlackRegion method or by directly setting the FocusStack.vfBlackTrace property. DefineBlackRegion allows a number of pixel indices to be provided that define a region in the stack which is expected to have zero fluorescence (for example, the interior of a blood vessel). This can be performed either by providing pixel indices, or through a GUI-based selection of a circular region.

Secondly, a baseline fluorescence distribution ($\hat{F}$_0_ and σ_*F*_0__) must be estimated and recorded for each frame. An example procedure for estimating the baseline distribution using FocusStack is given in Listing 1. In FocusStack, the baseline distribution is stored efficiently as stack meta-data (Figure 2). Each baseline frame is associated with a range of stack frames, leading to minimal storage requirements.

### 3.2. Stack segmentation, stimulus derandomization and extracting calcium responses

#### 3.2.1. Defining regions of interest (ROIs)

Since FocusStack objects appear as Matlab tensors, standard image-processing pipelines can be applied directly. We have included two simple pipelines: the first, FindCells_G, seek peaks of intensity in channel 1—useful for imaging with calcium indicators that brightly label cell nuclei; the second, FindCells_GR, subtracts channel 2 from channel 1—useful when a second channel is used for a neuron-excluding fluorescent label such as sulforhodamine. Code is also included to import ROI definitions from ImageJ (Schneider et al., 2012).

#### 3.2.2. Extracting calcium responses

In any good experiment design, stimulus presentation order is randomized. During analysis of the acquired time series data, stimulus segmentation, and derandomization therefore becomes an important but fiddly task. Our solution is to store the stimulus presentation order with the stack, along with information about stimulus duration, “blank” stimuli, and periods of stimulus presentation during which analysis of the calcium signals should be performed (see Figure 2).

Extracting calcium response time-series from a FocusStack object is accomplished using the ExtractRegionResponses function (see Listing 2). This workhorse function transparently performs stimulus derandomization, simultaneously averages and extracts responses from a number of arbitrary ROIs in the stack, segments the stack into single-trial per-neuron traces and returns estimated responses for each stimulus and each trial. FocusStack therefore provides an automated extraction of the peri-stimulus time histogram (PSTH) for each presented stimulus.

**Listing 2:**
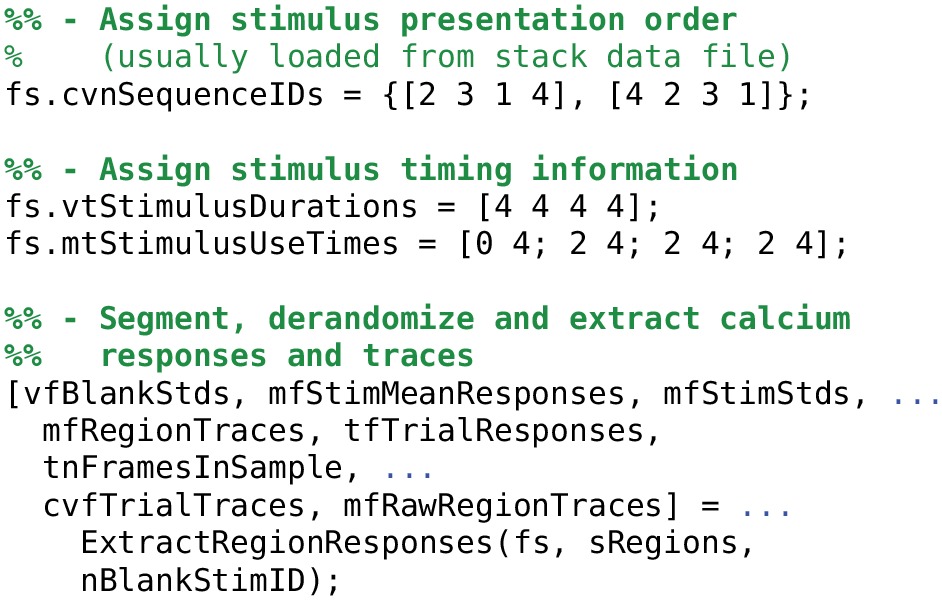
**Assigning stimulus durations and extracting derandomized calcium traces. Note that the order of stimulus presentation can and should be stored as meta-data by the two-photon acquisition system. If meta-data is present in the data files, then FocusStack will assign the meta-data to the stack when the stack is created**.

ExtractRegionResponses is highly modular, and allows the user to define what a “response” means for a given calcium trace. For example, toolbox functions are included to extract the mean, the peak, and the ratio of a calcium stack; all support either extraction of raw signals or Δ*F*/*F*_0_ processed data.

**Figure 3:**
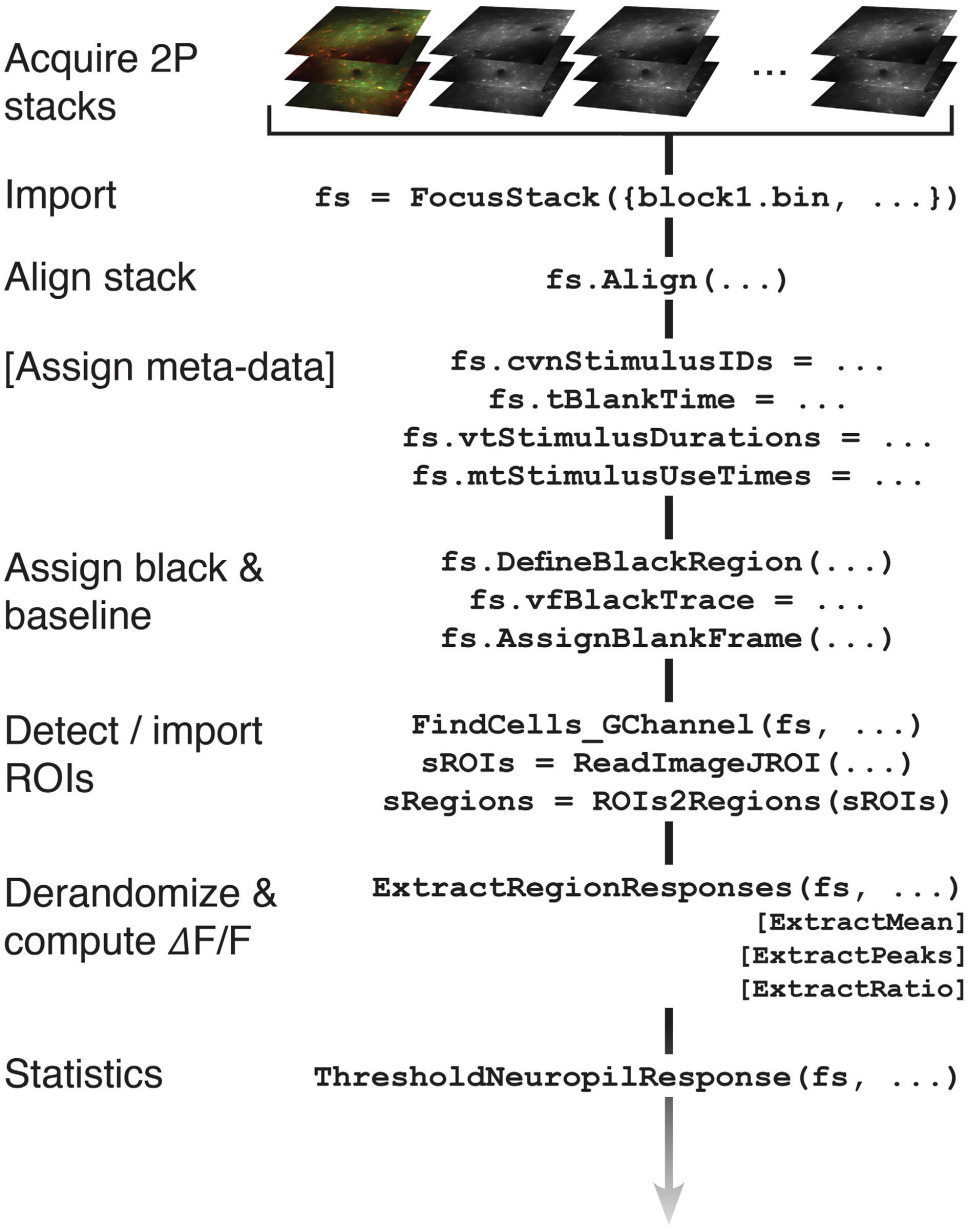
**Information flow of a FocusStack object, during standard analysis steps applied to a two-photon imaging stack**. Additional analysis steps can easily be added (see text).

A flowchart showing an example of information flow during standard two-photon analysis steps applied to a FocusStack object is given in Figure 3.

### 3.3. Interfacing with other software

ROIs are defined using the Matlab region structure format returned by bwconncomp. This means that FocusStack can easily accept ROI segmentations determined using the Matlab image processing toolbox. However, code is also included in FocusStack to import ROIs from ImageJ.

Since all responses traces and response values are produced by FocusStack in Matlab standard formats, existing software for processing calcium response traces can be used directly—for example, the fast non-negative deconvolution algorithm for estimating spike times of Vogelstein et al. (2010), the compressive-sensing approach of Dyer et al. (2013) or the peeling algorithm of Grewe et al. (2010) and Lütcke et al. (2013).

## 4. Low-level FocusStack representation

FocusStack already provides in-build access to the data format of a previously published 3D imaging software, “Focus” (Göbel et al., 2007) and the open source project “HelioScan” (Langer et al., 2013). There are presently a plethora of binary data formats in which two-photon imaging systems store recorded calcium signals. Many of these are *ad-hoc*, “in-house” formats, and which may change with little warning. For this reason, it is important that a general analysis toolchain is abstracted away from the particular binary format in which data is stored. We designed FocusStack such that the low-level representation is itself modular, with a standard interface to the rest of the FocusStack core code. This implies that adding support for a new data format is a matter of an hour's work or less, after which existing analysis scripts will run without modification.

FocusStack contains support for two low-level Matlab classes, which map binary data on disk to a Matlab tensor representation. The first, MappedTensor, handles arbitrary binary data files with linear representations and fixed numbers of bits per pixel. The second, TIFFStack, provides rapid access to standard multi-frame, multi-channel TIFF graphics files, which are generated by several common microscopy systems. Both classes use a lazy access paradigm, where data is only loaded from disk when needed.

### 4.1. MappedTensor class

The MappedTensor class^2^ transparently maps large tensors of arbitrary dimensions to temporary files on disk, or makes existing binary files available as Matlab tensors. Referencing is identical to a standard Matlab tensor, so a MappedTensor can be passed into functions without requiring that the function be written specifically to use MappedTensors. This is opposed to objects of the built-in Matlab class memmapfile, which cannot be used in such a way. memmapfile occasionally runs out of virtual addressing space, even if the data is stored only on disk. MappedTensor does not suffer from this problem. MappedTensors transparently support complex numbers, another advantage over memmapfile.

Being able to use MappedTensors as arguments to functions requires that the tensor is indexed inside the function (as opposed to manipulating the object without sub-referencing). This implies that a function using a MappedTensor must not be fully vectorized, but must operate on the mapped tensor in segments inside a for loop. Note that parfor loops are unsupported, but may work for local clusters or with a shared storage architecture. Functions that work on every element of a tensor, with an output the same size as the input tensor, can be applied to a MappedTensor without requiring the entire tensor to be allocated in memory. This is done with the convenience function SliceFunction.

MappedTensor offers support for basic operations such as permute and sum, without requiring space for the tensor to be allocated in memory. Many operations can be performed in *O*(1) time, such as negation, multiplication and addition, transpose and permute. Addition and subtraction of scalars are performed in *O*(*N*) time.

MappedTensor is implemented as a Matlab class, wrapping efficient file access and tensor handling functions. MappedTensor inherits from the Matlab handle class, which implies that duplicating a MappedTensor object does not duplicate the underlying data storage. Copies of a single MappedTensor contain the same data and properties, and modifying one copy modifies them all.

Examples of using MappedTensor objects are given in Listing 3.

**Listing 3:**
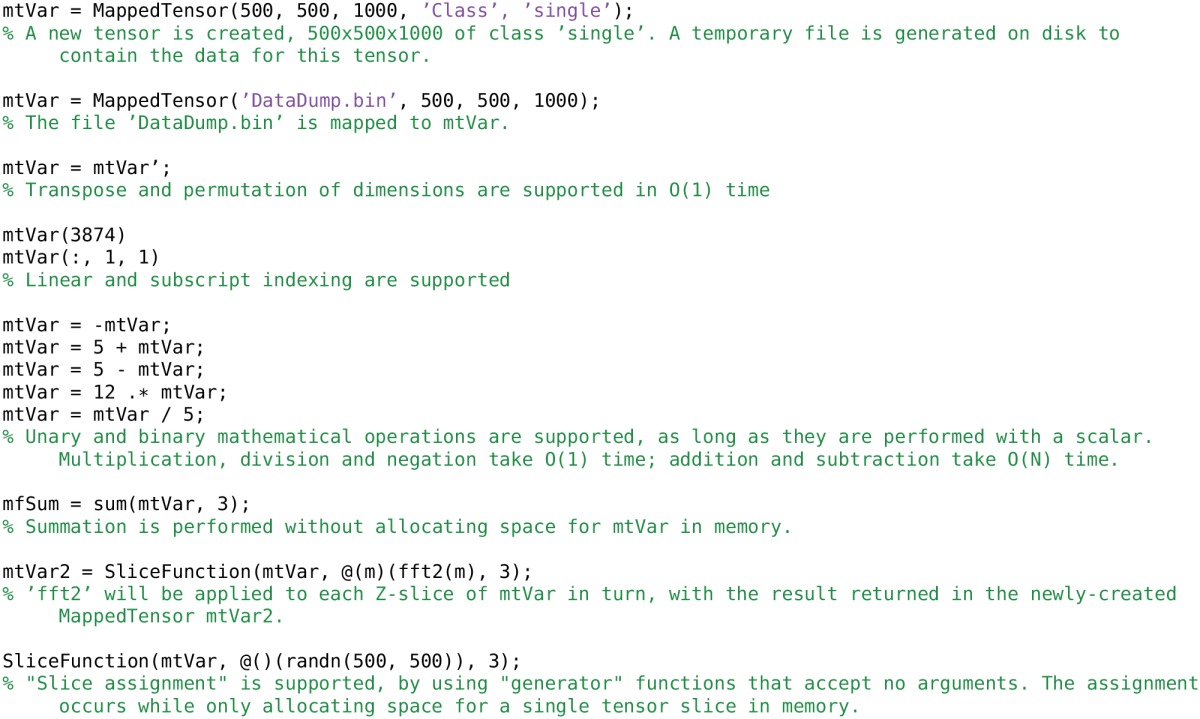
**Creating and accessing MappedTensor objects**.

### 4.2. TIFFStack class

A TIFFStack object^3^ behaves like a read-only memory mapped TIFF file. The entire image stack is treated as a Matlab tensor. Each frame of the file must have the same dimensions. Reading the image data is optimized to the extent possible; the header information is only read once. permute, ipermute and transpose are transparently supported, with *O*(1) time requirements.

Examples of using TIFFStack objects are given in Listing 4.

**Listing 4:**
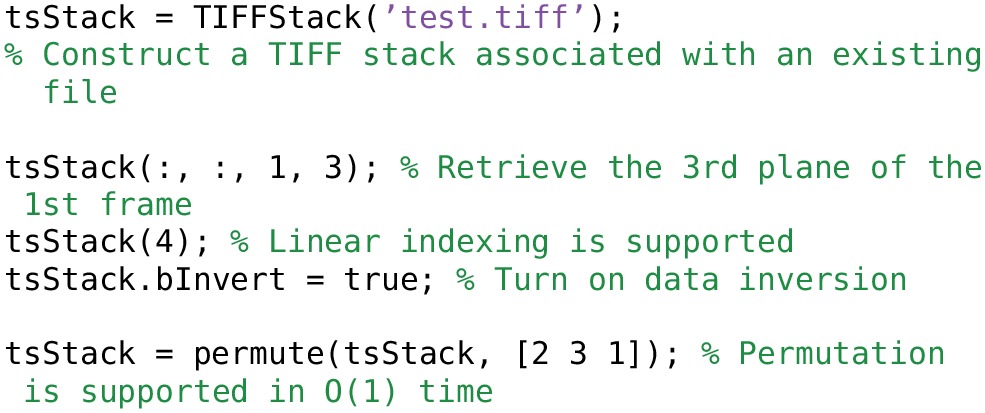
**Creating and accessing TIFFStack objects**.

### 4.3. Adapting FocusStack to new file formats

Enabling FocusStack to open new file formats requires adapting the FocusStack/OpenFiles static method. Depending on the file extension, OpenFiles must create a handle to a mapped file using MappedTensor, TIFFStack, memmapfile or any other appropriate method. OpenFiles must also extract any available meta-data concerning the stack, such as stimulus sequence and stack resolution. It is important for the design of FocusStack that binary data access be performed on a lazy basis, so that the memory footprint remains small.

The MappedTensor class described above is extremely flexible, and can easily be used to access binary data files with a wide range of formats.

### 4.4. Size and time benchmarks

Here we include some benchmarks for memory storage and data access time using FocusStack and TIFFStack, compared with loading stacks using the Matlab imread function and the Two-Photon Processor (2PP; Tomek et al., 2013). All benchmarks were performed on a MacBook Pro (two-core Intel Core i7 3 GHz; 8 GB RAM; SSD HD; OS X 10.0) running Matlab 2014a. Scripts used for time benchmarks of FocusStack and imread are shown in Listings 5 and 6; benchmark results are given in Table 3. Data storage requirements for FocusStack and TIFFStack objects were estimated by linearizing the objects using the Matlab struct command. When timing file loading, the range of several benchmark trials is reported, skipping the first trial.

**Listing 5:**
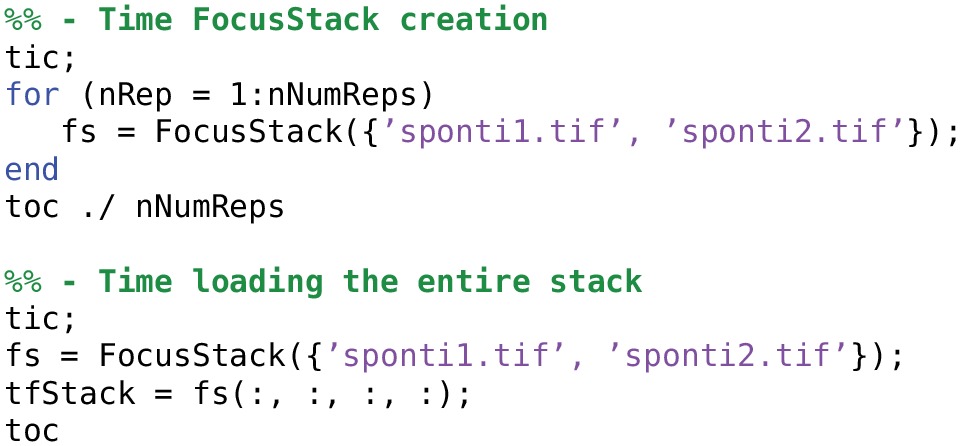
**Timing the loading of a stack using FocusStack**.

**Listing 6:**
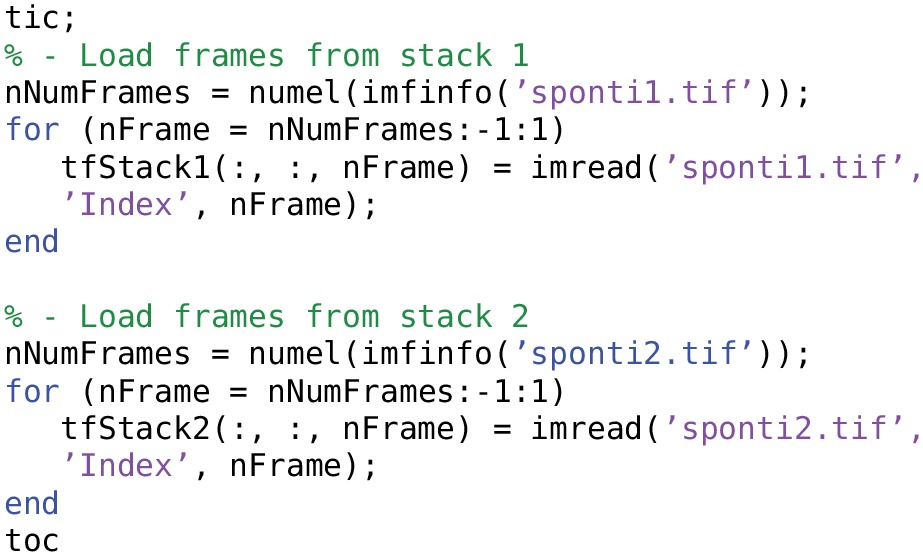
**Timing the loading of a TIFF file using imread**.

**Table 3:** **Memory storage and time benchmarks for FocusStack, TIFFStack, imread, and the Two-Photon Processor (2PP; Tomek et al., 2013)**.

**Benchmark**		**Result**
**BINARY FILE FORMAT**		
Data size on disk		241 MB^a^
Time to create FocusStack object		≈350 ms
Time to read in data for entire stack	**FocusStack**	16–17 s
Memory usage within Matlab	**FocusStack**	108 kB
	**data-native uint8** tensor	230 MB
	**default double** tensor	1.8 GB
**TIFF FILE FORMAT**		
Data size on disk		958 MB^b^
Time to create stack	**FocusStack**	≈280 ms
	**TIFFStack**	≈230 ms
Time to read in data for entire stack	**FocusStack**	18–25 s
	**TIFFStack**	6.5–7.4 s
	**imread**	12–17 s
	**Two-photon processor (2PP)**	57–68 s
Memory usage within Matlab	**FocusStack**	33 MB^c^
	**Two-photon processor (2PP)**	900 MB
	**data-native uint16** tensor	900 MB
	**default double** tensor	3.5 GB
**DATA ACCESS AND PROCESSING**		
Data size on disk		116 MB^d^
Time required to load data, align stack and extract calcium responses	**FocusStack**	150 s
	**Two-photon processor (2PP)**	470 s
Memory usage within Matlab	**FocusStack**	230 kB
	**Two-photon processor (2PP)**	116 MB

a 128 × 128 × 7378 × 2 pixels, 8-bit data across 7 files.

b 512 × 512 × 900 × 1 pixels, 16-bit data across 2 files.

c Memory usage by FocusStack for TIFF data is mostly consumed by caching of image header information within TIFFStack objects.

d 128 × 128 × 7378 × 1 pixels, 8-bit data across 7 files.

When accessing stacks stored in the “Focus” binary format, FocusStack required 0.05% of the memory storage than a Matlab matrix in a data-native format (uint8), and 0.006% of that required when using the default Matlab format (double).

When accessing data in TIFF format, FocusStack required 4% of the memory storage than using the 2PP or a data-native Matlab matrix (uint16), and 1% of that required when using the default Matlab format (double). In addition, TIFFStack and FocusStack were considerably faster when accessing data: 2PP required between three and nine times as long to read data. FocusStack and imread performed comparably, with FocusStack requiring 1.5 times as long as imread to read data; however, TIFFStack was approximately twice as fast as imread.

The low-level primitives used by FocusStack therefore allow efficient access to binary stack data, both in terms of speed and of memory usage. The time required to load data, align a stack and extract calcium responses was compared between FocusStack and 2PP. FocusStack/Align and FocusStack/ExtractRegionResponses were called in sequence to process a binary stack. The same stack was processed using 2PP via the TSeriesProcessor/getIntensities method, called with a minimal set of parameters. FocusStack completed alignment and signal extraction in only 30% of the time required by 2PP, and in 0.2% of the memory footprint. Note that the performance of both packages will depend greatly on the exact processing pipeline used.

## 5. High-level interface to stimServer

StimServer is a new, open source, Matlab-based stimulus generation and sequencing server for visual stimulation, using Psychtoolbox for low-level driving of a stimulus screen (Brainard, 1997; Pelli, 1997; Guizar-Sicairos et al., 2008). Stimuli are designed and configured on the server machine, after which StimServer is designed to be controlled remotely to initiate stimulus presentation. StimServer requires either the Matlab Instrument Control Toolbox (ICT^4^) or the TCP/UDP/IP Toolbox (PNET^5^; included with Psychtoolbox) for low-level network communication.

### 5.1. Configuring stimuli

An example of generating stimulus objects and configuring StimServer is given in Listings 7 and 8. Stimuli are represented as Matlab structures with a standard format that describes the parameters of a stimulus, which parameters are available for modification by the remote controlling process, and the names of the stimulus generation, presentation, and description functions.

**Listing 7:**
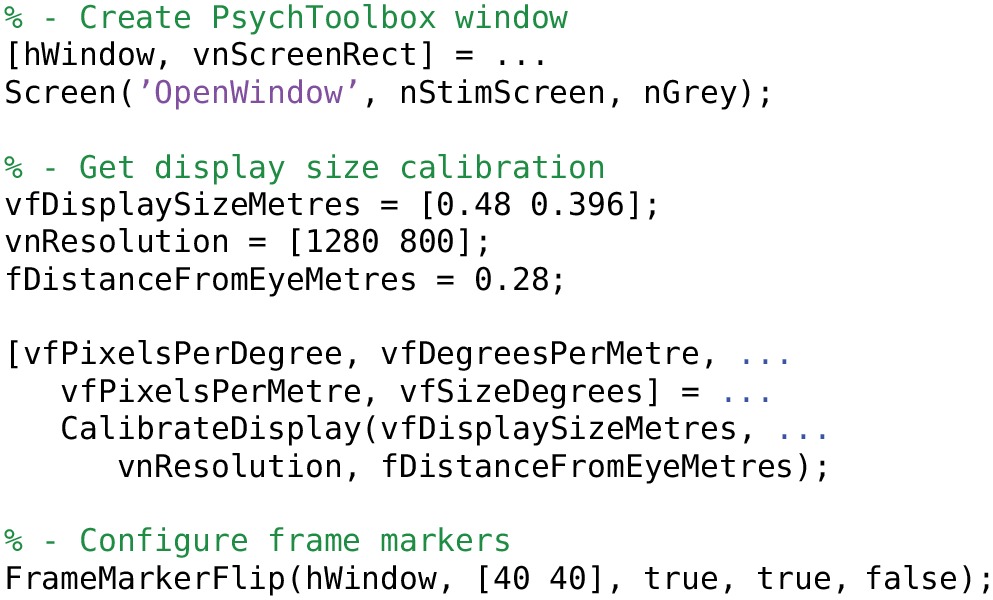
**Initialization of the StimServer environment**.

**Listing 8:**
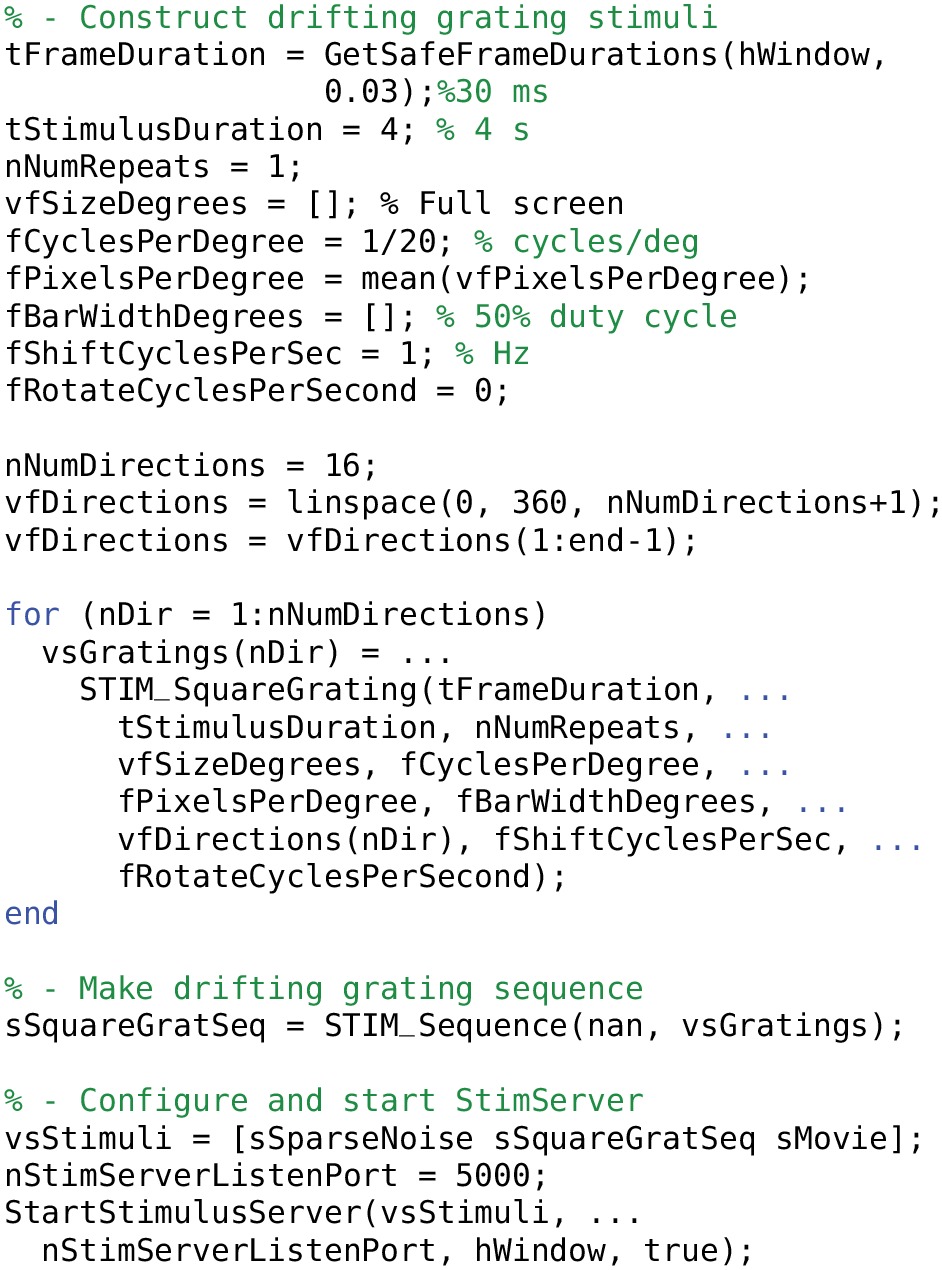
**Configuring a set of stimuli and starting the StimServer**.

Stimuli are configured using a set of generation functions (STIM…). These functions return stimulus objects which are then passed directly to StimServer. The number and identity of a set of stimuli is fixed once StimServer is started. However, many or all parameters of a given stimulus can be determined dynamically at presentation time, by sending a set of stimulus arguments over a network interface when triggering stimulus presentation. For example, the server could be configured with a single drifting grating stimulus. At presentation time, the network interface could dynamically set the orientation and drift speed, as well as other parameters of the stimulus.

### 5.2. Controlling the server remotely

**Figure 4:**
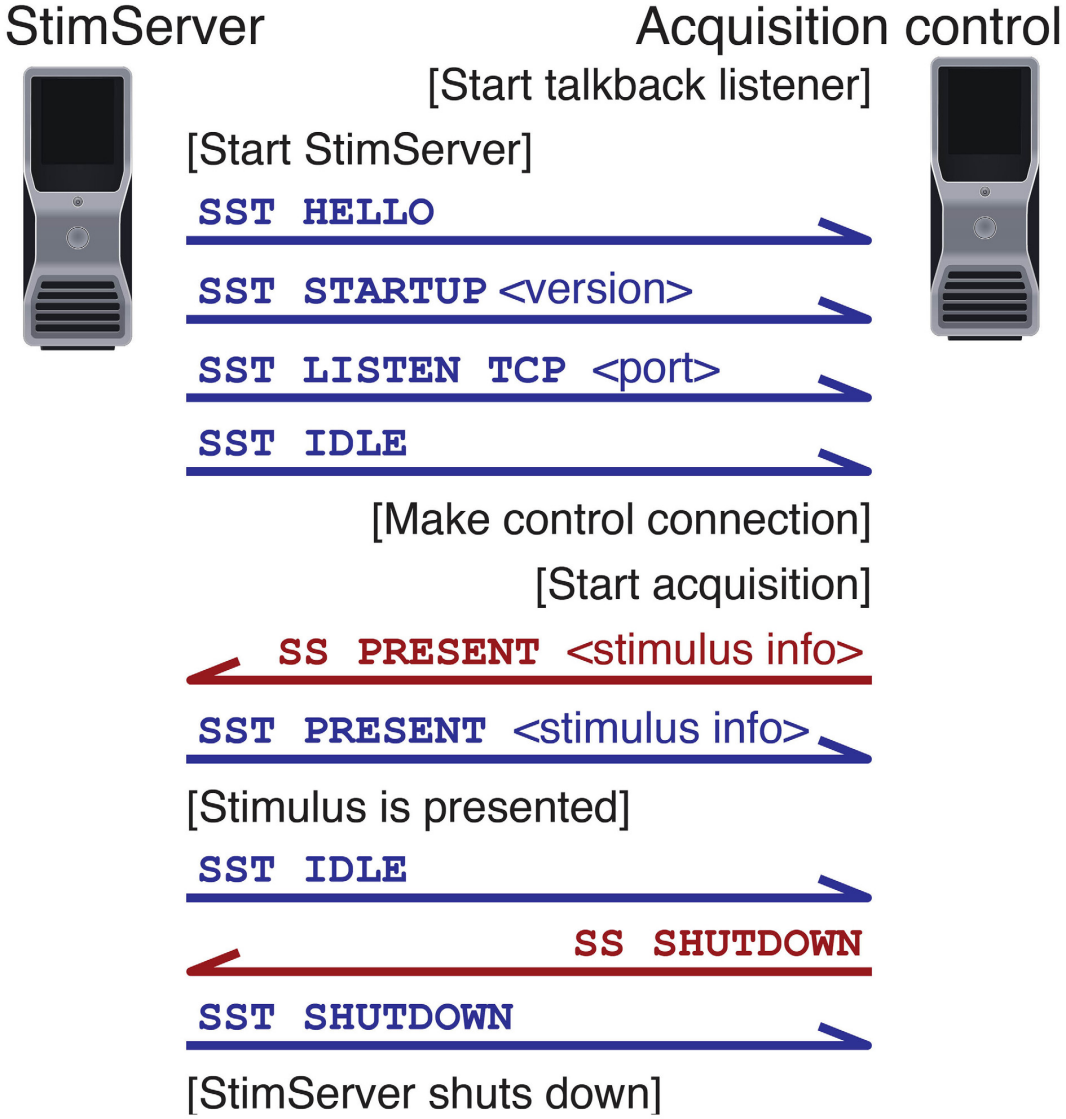
**Example dialogue between StimServer and controlling machine**. Commands are sent over the command channel (red); talkback notifications are sent of the talkback channel (blue).

Stimulus presentation is triggered over a network link (both TCP and UDP are supported). A series of textual commands are used to control stimulus presentation, parameters, and sequencing. An example dialogue between a controlling machine and StimServer is shown in Figure 4.

Most parameters of a visual stimulus can be controlled remotely at presentation time, including the order of presentation of a stimulus sequence. In this case the remote controlling process generates a pseudo-random sequence in which to present a set of stimuli; this sequence can then be recorded as meta-data along with the acquired neuronal responses. Alternatively, dynamic stimulation can be performed—for example, setting an arbitrary orientation or spatial frequency of a drifting grating—online during an experiment.

Once a connection is established with the server, a reverse “talkback” connection can be configured so that feedback and confirmation of commands is available to the remote controlling process. Logging of stimulus commands and any error messages are either logged by StimServer locally to a file, or delivered over another network connection for storage along with the acquired experimental data.

### 5.3. Adding new stimuli

Many useful visual stimuli are available out of the box (see Table 4), and including new stimuli is straightforward due to the modular architecture of StimServer. Stimuli have a common defining structure, requiring a generation function, a description function and a presentation function. Any new stimulus that adheres to this interface can then be included in new stimulus sets, transparently to the core StimServer code.

**Table 4:** **Stimuli provided out of the box by StimServer**.

**Stimulus (STIM_…)**	**Description**
Blank	Blank stimulus
Sequence	Group a set of other stimuli into a randomizable sequence
SineGrating	Drifting and rotating masked sinusoidal grating
SinePlaid	Drifting and rotating plaid composed of two additively combined sinusoidal gratings, with arbitrary relative orientations
SquareGrating	Drifting and rotating masked square-wave grating
SquarePlaid	Drifting and rotating plaid composed of two additively combined square-wave gratings, with arbitrary relative orientations
OscillatingGrating	Static oriented square-wave grating that oscillates in contrast
OscillatingPlaid	Static plaid composed of two oriented square-wave gratings that oscillate in contrast and phase
SparseNoise	Sparse noise composed of pixels arranged in a grid
SparseNoiseFlicker	Sparse noise composed of pixels that oscillate in contrast
SparseGrating	Sparse noise, where each pixel is a masked square-wave grating that drifts and rotates
BandLimitedNoise	Spatially- and temporally-filtered white noise
DotKinematogram	Random dot kinematogram stimulus
FlashedImageSequence	A sequence of flashed arbitrary images
GaborField	A field of Gabors with arbitrary locations and arbitrary individual parameters, that drift in phase and rotate
GaborGrid	A regular grid of Gabors with arbitrary individual parameters, that drift in phase and rotate
MaskedMovie	Present an arbitrary movie from a file, with a circular mask

StimServer also provides a PresentSimpleStimulus function, which takes care of all the low-level timing and presentation tasks for stimuli comprising drifting and rotating textures, with optional masking.

**Figure 5:**
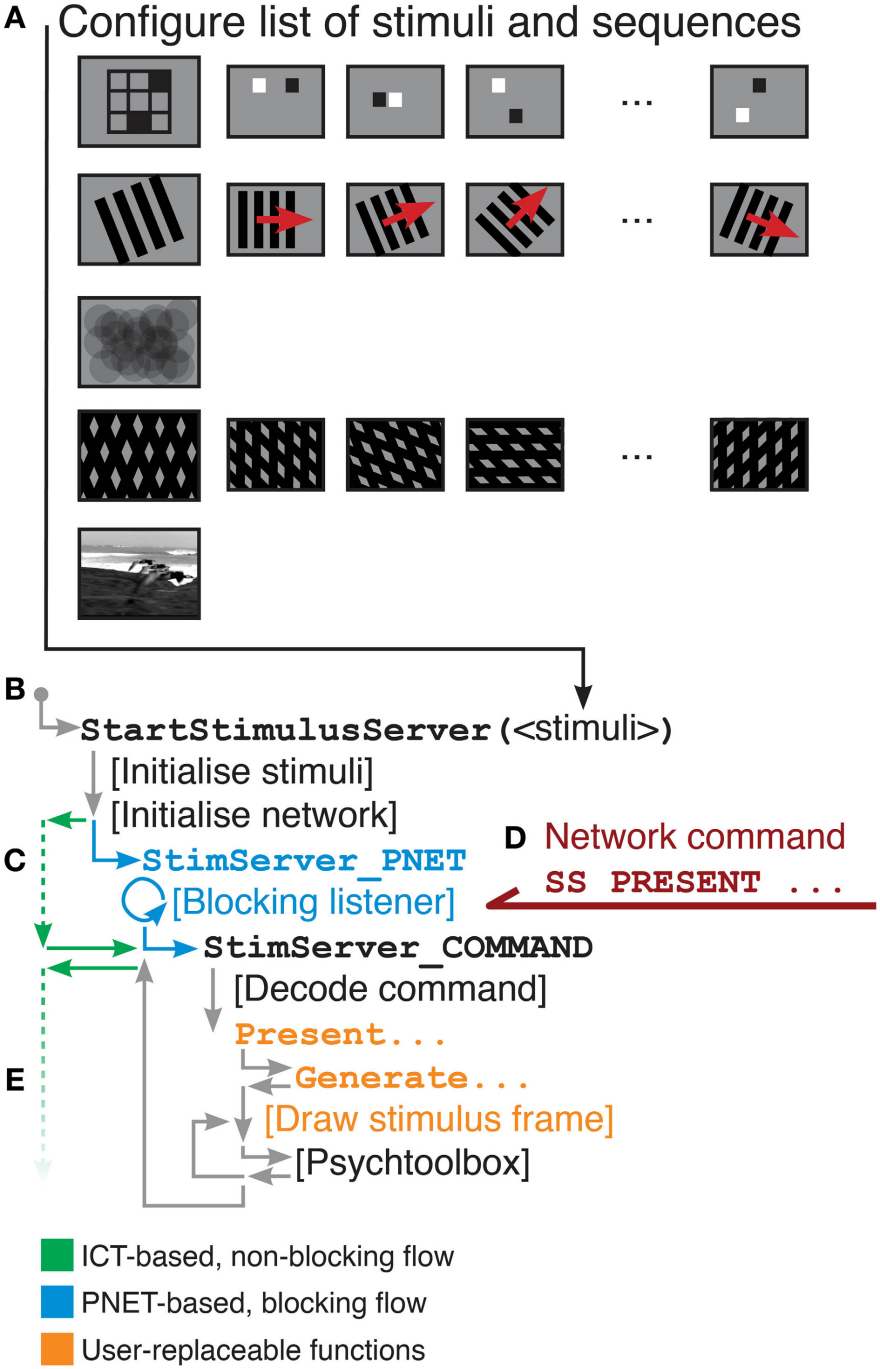
**Overview of StimServer information flow. (A)** A list of stimuli and stimulus sequences is constructed (see Listing 8). **(B)** StartStimulusServer is called from the Matlab command line. **(C)** If the Instrument Control Toolbox is used for network communication (green), control returns to the Matlab command line (i.e., non-blocking network listening). If PNET is used for network communication then StimServer enters a blocking poll loop (blue). When a presentation command is received **(D)**, the stimulus-defined presentation function is called **(E)**. Commands shown in orange are modular, and can be replaced to introduce new stimulus classes.

A flowchart showing execution flow through StimServer, indicating functions replaced by user-defined stimuli, is given in Figure 5.

## 6. Example experiments and analysis

In this section we present analysis of *in vivo* two-photon calcium imaging recordings from mouse primary visual cortex (V1). The goal of the experiment was to characterize responses in mouse V1 to drifting grating and to natural visual stimuli, in populations of neurons with overlapping receptive fields. Experimental procedures followed the guidelines of the Veterinary Office of Switzerland and were approved by the Cantonal Veterinary Office in Zurich.

Example data and example scripts that reproduce the analyses in this section are available as supplementary information.

### 6.1. Two-photon calcium imaging of neuronal responses in mouse V1

Methods for two-photon acquisition were as described elsewhere (Kampa et al., 2011; Roth et al., 2012). Briefly, C57BL/6 mice (at P75–P90) were initially anesthetized with 4–5% isoflurane in O2 and maintained on 1.5–2% during the surgical procedure. The primary visual cortex (V1) was localized using intrinsic imaging. A craniotomy of 3–4 mm was opened above the region of strongest intrinsic signal response. The genetically encoded calcium indicator GCaMP6m (Chen et al., 2013) (AAV1.Syn.GCaMP6m.WPRE.SV40; UPenn) was injected around 250 μ m below the cortical surface to target superficial layer neurons. The craniotomy was then sealed with a glass window. After recovery and expression of the calcium indicator, animals were head-fixed and calcium transients were acquired using a custom-built two-photon microscope equipped with a 40× water-immersion objective (LUMPlanFl/IR, 0.8 NA; Olympus). Frames of 128 × 128 pixels were acquired at 7.81 Hz with bidirectional scanning using custom-written software (“Focus”; LabView; National Instruments).

Visual stimuli generated with StimServer were presented on a 24 inch LCD monitor (1200 × 800 pixels; 60 Hz) to the left eye of the mouse, spanning approximately 80 visual degrees. Details of each visual stimulus are given below.

### 6.2. Receptive field localization

Knowing the location in visual space of the receptive fields (RF) of the neurons in an imaged region of visual cortex is important, if properties of the neural responses should be compared between neurons with fully overlapping RFs. If masked stimuli are to be used, the location and extent of the mask will depend also on the RF locations of the recorded neurons.

StimServer provides several stimuli for estimating RF locations: STIM_SparseNoise uses flashed high-contrast squares; STIM_SparseNoiseFlicker uses contrast-reversing squares; and STIM_SparseGrating uses patches of drifting and rotating high-contrast gratings. We configured a 5×5 grid of 12 deg. diameter pixels on the stimulus screen, with 40% overlap between adjacent pixels. Each pixel contained a 100% contrast vertical square-wave grating of 25 deg. per cycle, drifting at 1 Hz and presented for 1 s, with the full set of pixels presented in random order. Seven random repeats of the stimulus were collected to estimate RF location.

**Figure 6:**
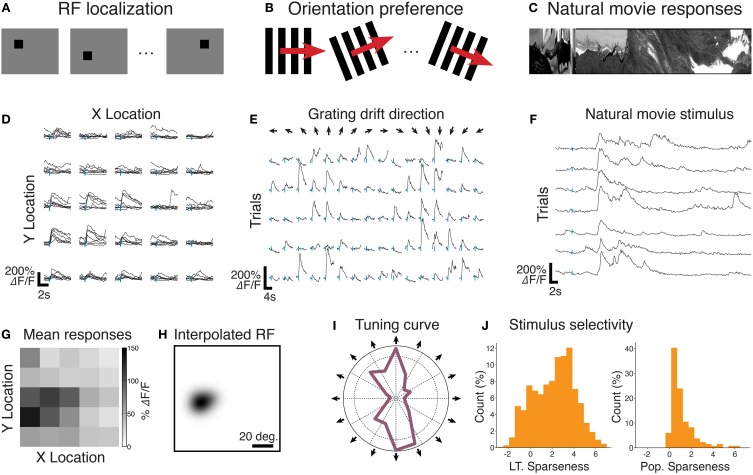
**Example analyses conducted with FocusStack and StimServer from recordings made in mouse V1. (A)** An RF localization experiment, where a sparse random stimulus is presented over a 5×5 mesh. **(B)** Measuring preferred orientation using drifting gratings. **(C)** Recording single-neuron and population responses to natural movie stimuli. **(D)** Single-trial single-neuron calcium responses to sparse noise stimuli. **(E)** Single-trial single-neuron responses to drifting high-contrast gratings. **(F)** Single-trial single-neuron calcium responses to a natural movie stimulus. **(G)** and **(H)** show the estimated RF location for the neuron shown in **(D)**. **(I)** The trial-averaged direction tuning curve for the neuron shown in **(E)**. **(J)** The population distribution of lifetime (L.T.) and population (Pop.) sparseness, from responses imaged simultaneously with the neuron shown in **(F)**. Stimulus onset in all traces **(D, E, F)** is indicated by a vertical tick mark. Data provided by M. Roth.

An example of RF localization analysis is given in Figure 6. Segmented single-trial per-pixel responses are shown in Figure 6D; the trial-averaged response matrix is shown in Figure 6G. Both come directly from ExtractRegionResponses. A smoothed RF estimate was obtained by summing Gaussian fields located at each pixel, with a diameter of 12 deg., modulated by the amplitude of the average calcium response of that pixel (Figure 6H).

### 6.3. Orientation tuning

The canonical cortically-derived feature in primary visual cortex is tuning for the orientation (or direction) of a drifting edge (Hubel and Wiesel, 1962; Ohki et al., 2005). We used responses to drifting grating stimuli to characterize the direction tuning curves of neurons in mouse V1.

StimServer provides drifting sinusoidal and drifting square-wave grating stimuli with a large range of manipulatable parameters. We presented full-field drifting high-contrast sinusoidal gratings at 16 drift directions, with spatial frequency of 20 deg per cycle and temporal frequency of 1 Hz (the STIM_SineGrating stimulus provided by StimServer). These stimuli were presented for 2 s each in random order, over 5 trials.

An example analysis of orientation tuning of a single cortical neuron is given in Figure 6. Segmented single-trial single-neuron responses are shown in Figure 6E. A polar plot of the trial-averaged responses for the same neuron are shown in Figure 6I. Both these data come directly from ExtractRegionResponses.

### 6.4. Natural movie representations

Neurons in visual cortex show complex selectivity for natural scenes and movies (Kampa et al., 2011). We recorded the responses of populations of neurons in mouse V1, to a sequence of short grayscale movies with normalized contrast. We characterized the efficiency of encoding natural movies on a single-neuron level and on a population level, by measuring the sparseness of neuronal responses.

StimServer provides stimuli for presenting randomized sequences of flashed images (STIM_FlashedImageSequence), as well as efficient stimulation with movies in standard Matlab-readable formats (STIM_MaskedMovie). Both these stimuli attempt to cache frames to the extent possible, leading to efficient presentation of stimuli without dropped frames. We presented 7 trials of a 43 s duration natural movie sequence (30 Hz movie frame rate), centered at the average location of the RF of the imaged population, and spanning approximately 70 visual degrees. The movie consisted of a sequence of three segments of video. Responses up to 1.5 s post the onset of the stimulus and after each movie transition were excluded from analysis.

An example analysis of the natural movie response of a single neuron in mouse V1 is given in Figure 6. Segmented single trial responses are shown in Figure 6F. Once again, these traces come directly from ExtractRegionResponses. An analysis of lifetime (LT) and population (Pop.) response sparseness, defined as the skewness of the calcium responses either over time (LT) or over simultaneous responses in the population (Pop.), is shown in Figure 6J. These data were calculated simply by taking the trial-averaged response matrix from ExtractRegionResponses, and passing it to the Matlab skewness function.

## 7. Conclusion

FocusStack provides a toolbox for simple yet powerful analysis of calcium imaging data. It presents an abstraction layer that takes advantage of standard Matlab tensor representations, but facilitates analysis by being aware of stimulus information and other experiment-related meta-data required to interpret neuronal responses (Figure 2). Many low-level, repeated tasks of calcium signal extraction and analysis are taken care of by the toolbox, ensuring consistent analysis between experiments and minimizing errors introduced by re-writing code.

StimServer provides a modular toolbox for stimulus generation and sequencing in Matlab, in conjunction with Psychtoolbox. It is designed to integrate into two-photon imaging systems, by allowing triggering of arbitrary stimuli over a network interface (Figures 1, 4, 5). Presentation order and most stimulus parameters can be reconfigured dynamically over the network interface during an experiment, allowing a two-photon acquisition system to sequence visual stimuli and then store stimulus information along with acquired imaging data.

When this stimulus meta-data is provided to FocusStack, the toolbox takes care of extraction of stimulus-related responses, by automatically performing time-series segmentation and derandomization of a two-photon stack. This implies that responses to complex and arbitrary sets of stimuli can be extracted and analyzed easily with few lines of code (see Figure 6).

FocusStack and StimServer comprise an open-source toolchain provided to the neuroscience community. We expect that the open availability and easy to use structure will encourage uptake of consistent analysis tools in the field, as well as many contributions to add and exchange features in both toolboxes.

FocusStack and StimServer are available as version-controlled GIT repositories, or as stand-alone downloads, from https://bitbucket.org/DylanMuir/twophotonanalysis and https://bitbucket.org/DylanMuir/stimserver.

## Author contributions

Dylan R. Muir designed and implemented the toolbox code, and wrote the manuscript. Björn M. Kampa contributed to the toolbox code, and wrote the manuscript.

## Funding

This work was supported by the Novartis Foundation (grant to Dylan R. Muir), Velux Stiftung (grant to Dylan R. Muir), the Swiss National Science Foundation (Grant Nr. 31-120480 to Björn M. Kampa), and the EU-FP7 program (BrainScales project 269921 to Björn M. Kampa).

### Conflict of interest statement

The authors declare that the research was conducted in the absence of any commercial or financial relationships that could be construed as a potential conflict of interest.

## Footnotes

^1^http://dylan-muir.com/articles/mapped_tensor/

^2^http://dylan-muir.com/articles/tiffstack/

^3^http://www.mathworks.com/products/instrument/

^4^http://www.mathworks.com/matlabcentral/fileexchange/345-tcp-udp-ip-toolbox-2-0-6
