# Supplementary material for: FocusStack and StimServer: a new open source MATLAB toolchain for visual stimulation and analysis of two-photon calcium neuronal imaging data
Source: Front Neuroinform. 2015 Jan 20;8:85. doi: 10.3389/fninf.2014.00085 (PMC4299654; doi:10.3389/fninf.2014.00085)
Supplement: Supplementary file 1 [file DataSheet1.PDF]

Frontiers in Neuroinformatics

Methods
18 November 2014

Supplementary Material: FocusStack and StimServer: A new open source MATLAB toolchain for visual stimulation and analysis of two-photon calcium neuronal imaging data

Dylan Richard Muir [1,2,*], **Björn M. Kampa** [1,3]

[1] *Department of Neurophysiology, Brain Research Institute, University of Zürich, Zürich, Switzerland*
[2] *Biozentrum, University of Basel, Basel, Switzerland*
[3] *Department of Neurophysiology, Institute of Biology 2, RWTH Aachen University, Aachen, Germany*

Correspondence*:
Dylan Richard Muir
Biozentrum, University of Basel, Klingelbergstrasse 50/70, 4056 Basel, Switzerland, dylan.muir@unibas.ch

1 EXAMPLE CODE

The code used to generate the panels in Figures 2 and 6 is included as supplementary material, in the file `GenerateAllFigurePanels.m`.

An example script to configure and start `StimServer` is included as supplementary material, in the file `ExampleStimServerScript.m`.

2 SUPPLEMENTARY DATA

The binary source data from several two-photon calcium imaging sessions is included as supplementary material. These source files were analysed to generate the panels shown in Figures 2 and 6. Please download the data from `http://dylan-muir.com/resources/data/FS_SS_ SupplementaryData.zip`
